# Supplementary material for: Genome-Wide Association Study Reveals Host Factors Affecting Conjugation in Escherichia coli
Source: Microorganisms. 2022 Mar 12;10(3):608. doi: 10.3390/microorganisms10030608 (PMC8954029; doi:10.3390/microorganisms10030608)
Supplement: Supplementary file 1 [file microorganisms-10-00608-s001.zip › microorganisms-1596076-supplementary/Figures_S1-10.pdf]

Supplementary Figures

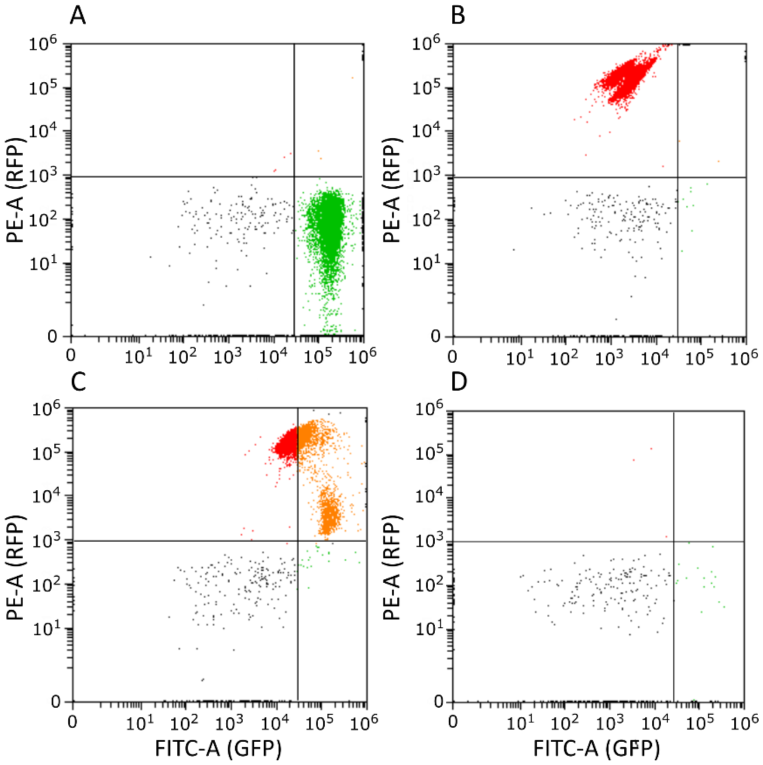

Figure S1. Controls in the cytometric assay for quantifying conjugation efficiencies. Thresholds were chosen based on the signal produced by the donor BW25113 pKJK10 (**A**), the recipient BW25113 pSB1C3-mRFP1 (**B**) and the transconjugant BW25113 pSB1C3-mRFP1 pKJK10 (**C**). The majority of the background noise is non-fluorescent (**D**).

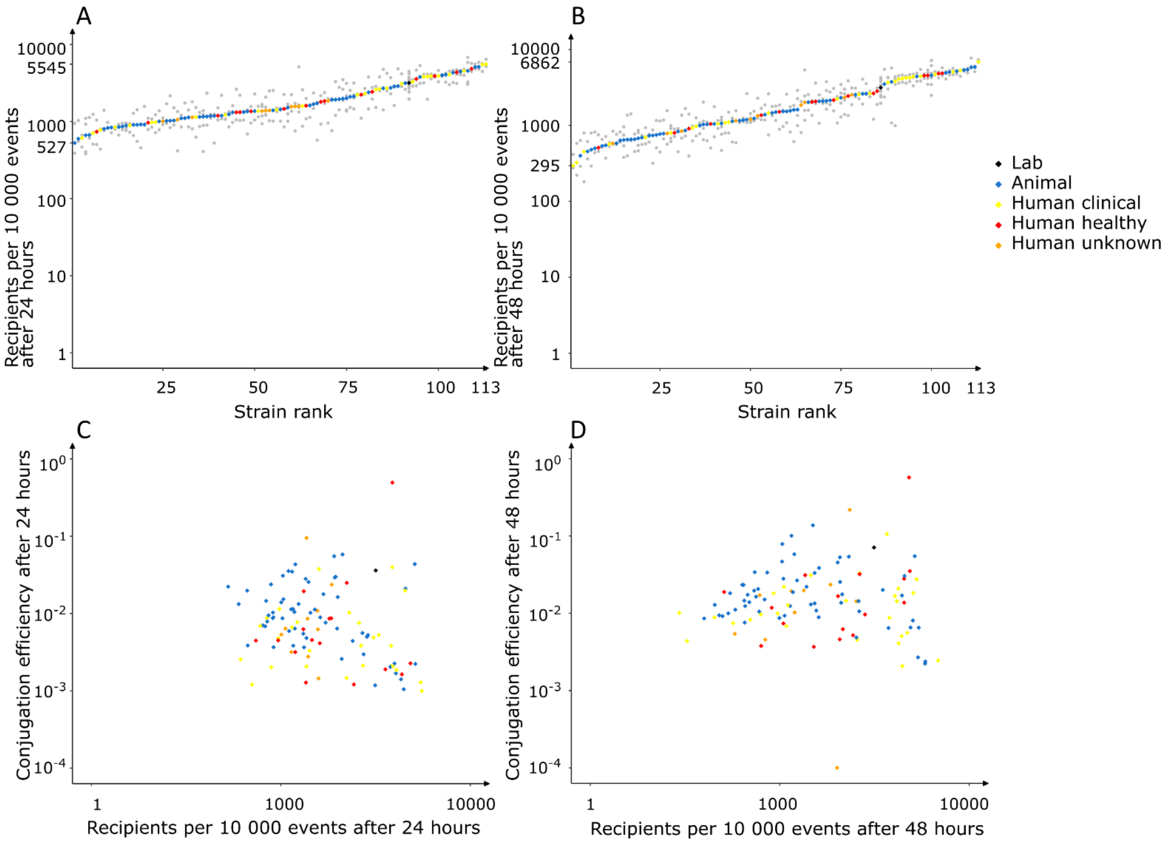

Figure S2. Effect of the recipient fraction on conjugation efficiency. The fraction of recipients varies with the used donor strain after 24 hours (**A**) and after 48 hours (**B**). **C**) After 24 hours a weak negative correlation is observed between the conjugation efficiency [transconjugants/recipient] and the fraction recipients [recipients/10 000 events] ($r_{Spearman}$ = -0.24, p = 0.001). **D**) After 48 hours, no correlation is observed between the conjugation efficiency [transconjugants/recipient] and the fraction recipients [recipients/10 000 events] ($r_{Spearman}$ = 0.01, p = 0.941).

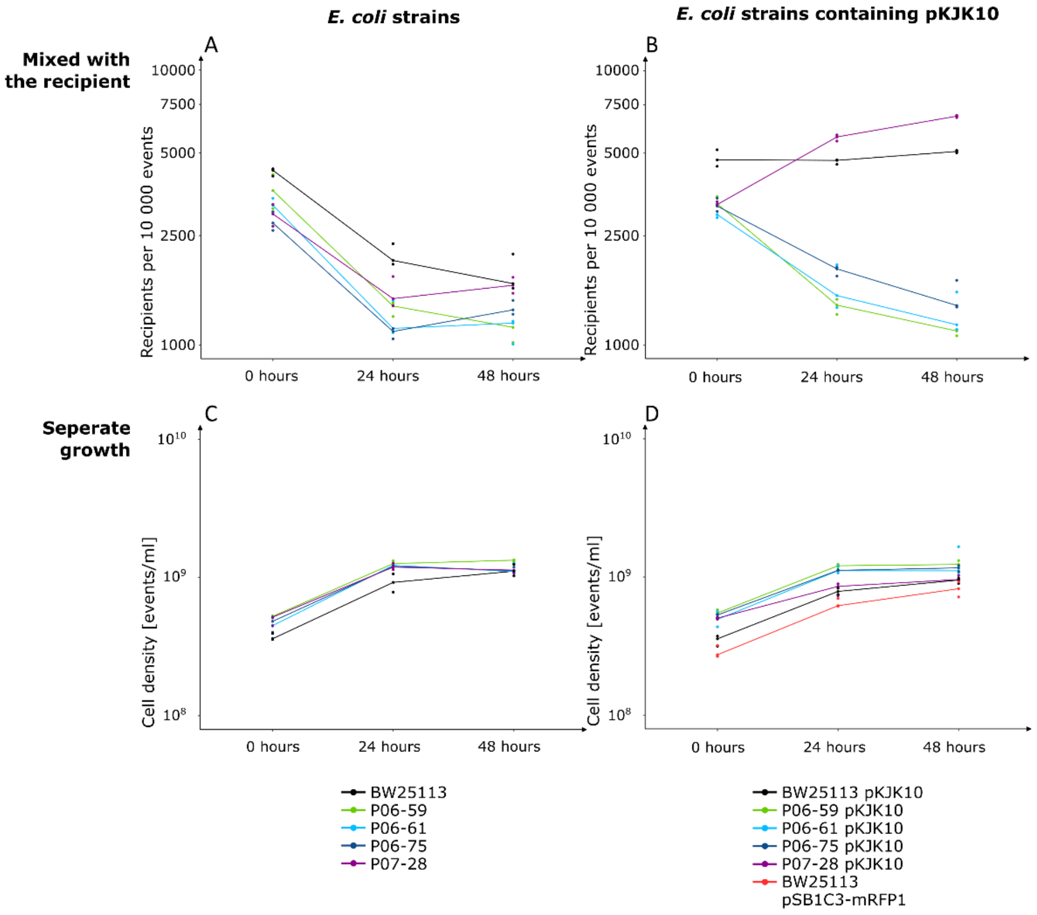

Figure S3. Competition between a selection of *E. coli* strains and BW25113 pSB1C3-mRFP1. The fraction of BW25113 pSB1C3-mRFP1 cells, referred to as the recipient, was quantified for *E. coli* strains without pKJK10 in competition with BW25113 pSB1C3-mRFP1 (**A**) and *E. coli* strains containing pKJK10 in competition with BW25113 pSB1C3-mRFP1 (**B**). Cells were counted by flow cytometry after 0 hours, 24 hours, and 48 hours. A Dunn's test and a Conover test demonstrated that the recipient fraction significantly differed between 0 hours and 24 hours, between 0 hours and 48 hours, or between 0 hours and 24 and 48 hours for all strains except P06-61 and BW25113 pKJK10. In addition, the growth of all strains was measured in absence of BW25113 pSB1C3-mRFP1 (**C, D**). A Dunn's test and a Conover test demonstrated a significant difference between 0 hours and 24 hours, between 0 hours and 48 hours, or between 0 hours and 24 and 48 hours for all strains except P06-61 pKJK10. To conclude, all strains except P06-61, P06-61 pKJK10 and BW25113 pKJK10 showed an increase in cell density when incubated in absence of BW25113 pSB1C3-mRFP1, and an increase or decrease in recipient fraction when incubated in presence of BW25113 pSB1C3-mRFP1. This points towards competition.

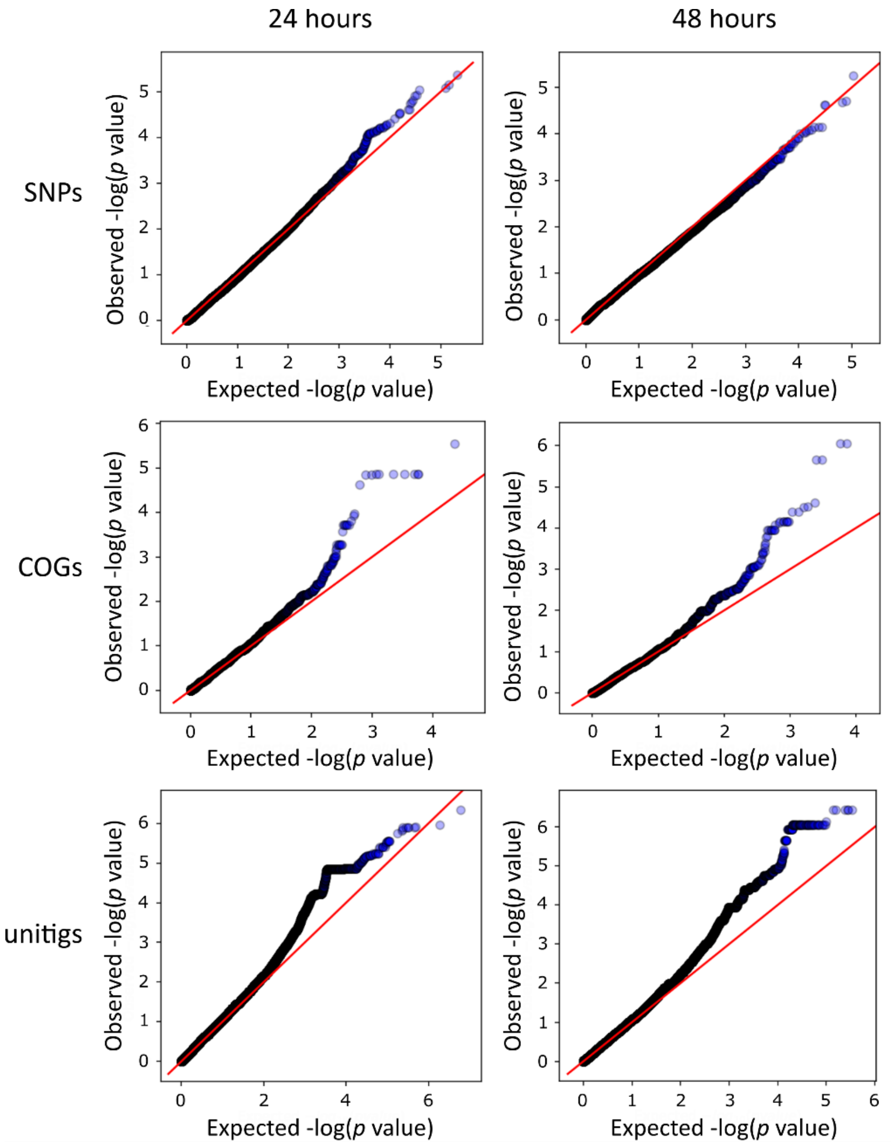

Figure S4. Quantile-quantile (QQ) plot of the -log(*p* values) obtained by SNP, COG, unitig association with the log-transformed conjugation efficiency after 24 hours and 48 hours using the linear mixed model. The y = x reference line is indicated in red.

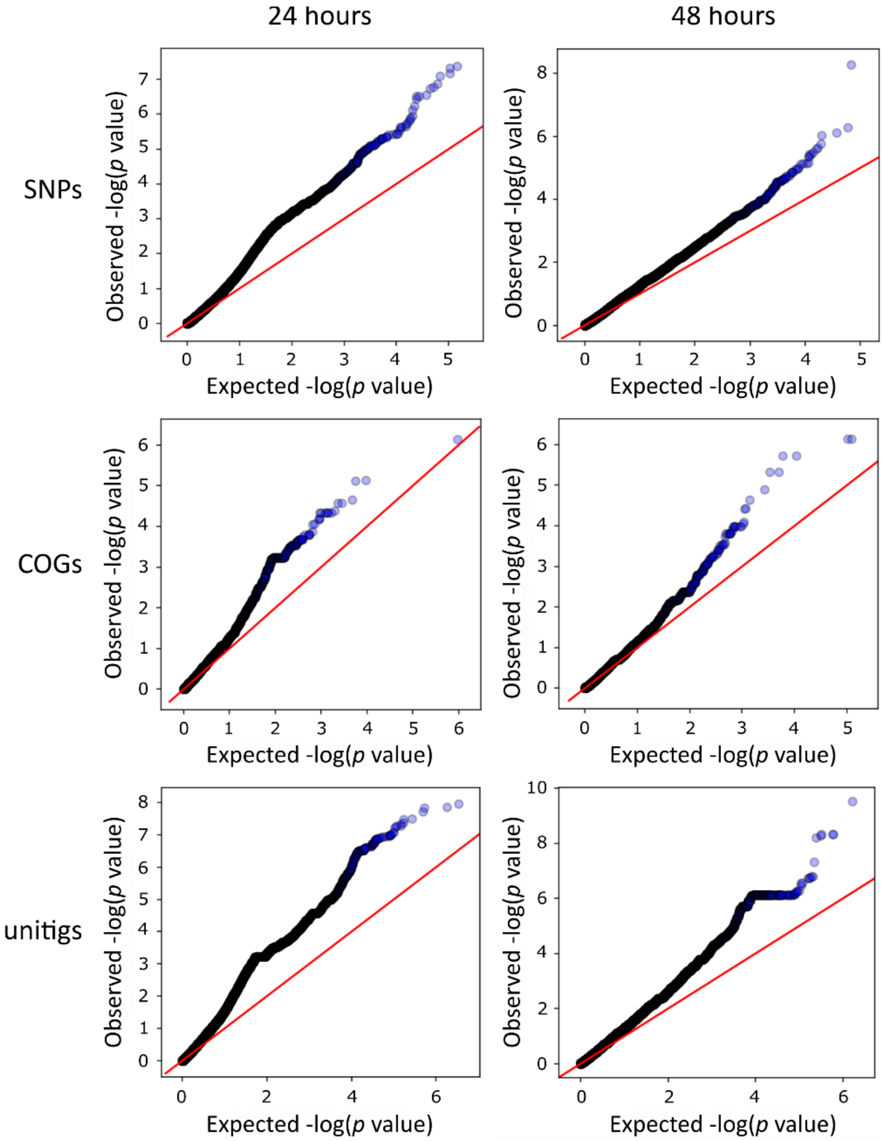

Figure S5. Quantile-quantile (QQ) plot of the -log(*p* values) obtained by SNP, COG, unitig association with the log-transformed conjugation efficiency after 24 hours and 48 hours using the fixed effects model. The y = x reference line is indicated in red.

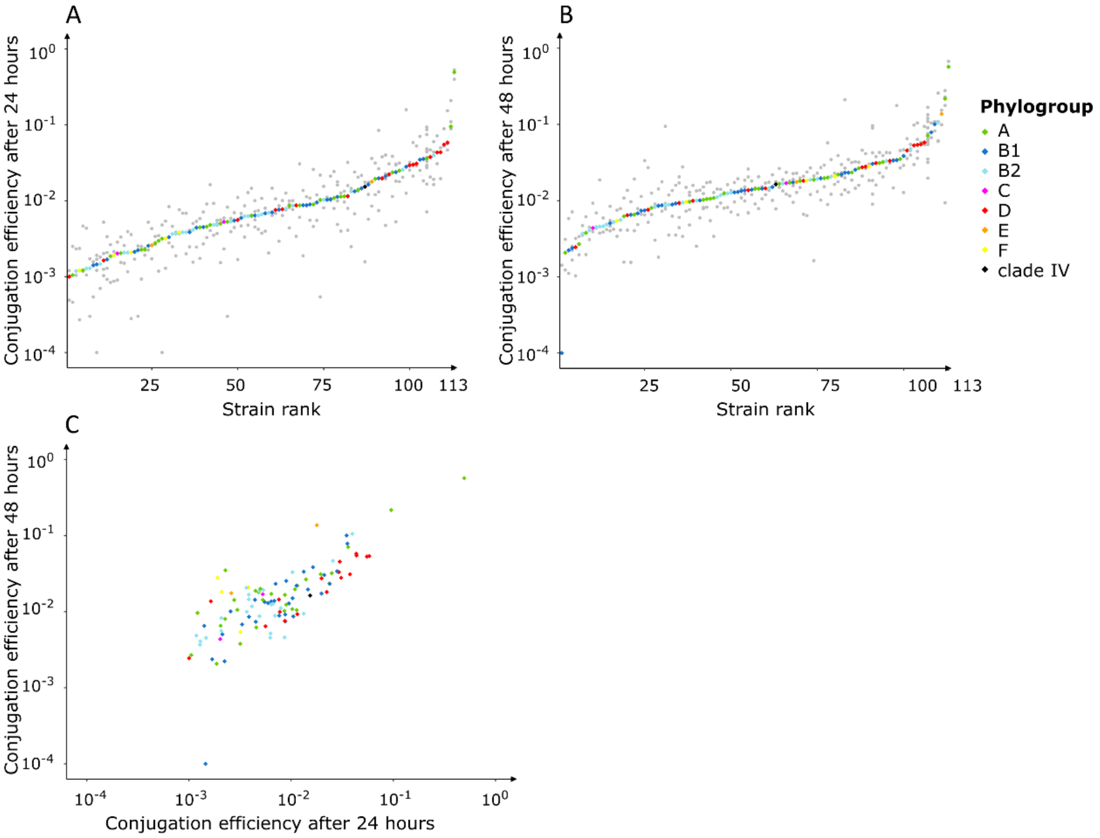

Figure S6. Effect of phylogroup on conjugation. Diverse *E. coli* strains were screened as donors in conjugation. The conjugation efficiency was quantified after 24 hours (**A**) and 48 hours (**B**). Per strain three biological replicas (grey) were tested and the median was calculated (colored diamonds). A Kruskal-Wallis test demonstrated that the effect of phylogroup on conjugation efficiency was significant after 24 hours and not significant after 48 hours (p values 4.1 x 10^{-3} and 2.1 x 10^{-1}, respectively).

Phylogroup	Sample size	Median conjugation efficiency after 24 hours	Median conjugation efficiency after 48 hours
A	29	0.00639	0.01670
B1	32	0.00818	0.01350
B2	25	0.00490	0.00998
C	2	0.00364	0.01070
D	17	0.02230	0.02740
E	2	0.01020	0.07750
F	5	0.00209	0.01830
clade IV	1	0.01530	0.01640

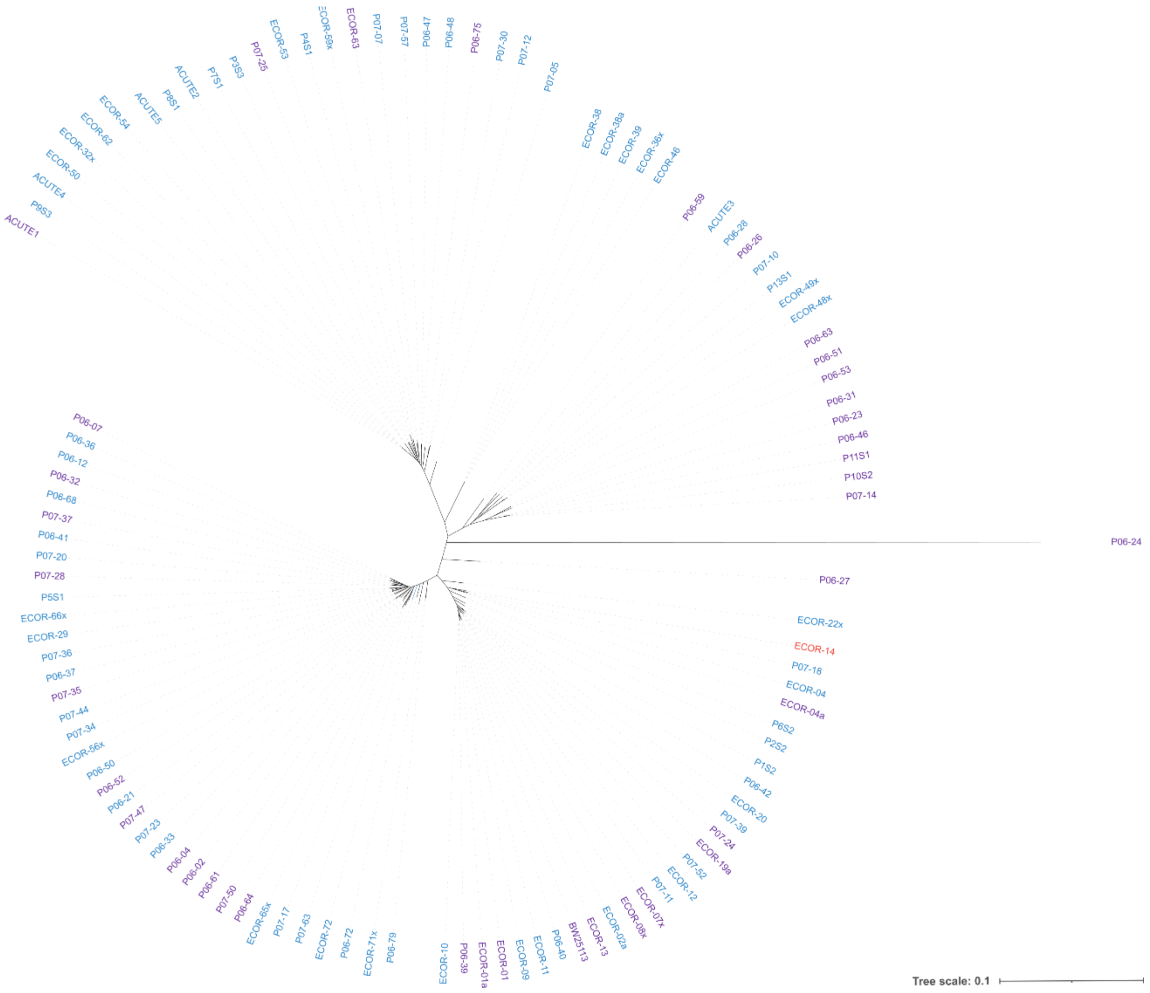

Figure S7. Conjugation efficiencies after 24 hours indicated on the RAxML phylogenetic tree. The labels of the strains are colored red for conjugation efficiencies between 10^0 and 10^{-1}, purple for conjugation efficiencies between 10^{-1} and 10^{-2}, and blue for conjugation efficiencies between 10^{-2} and 10^{-3}. Figure was created in iTOL 6.5.2.

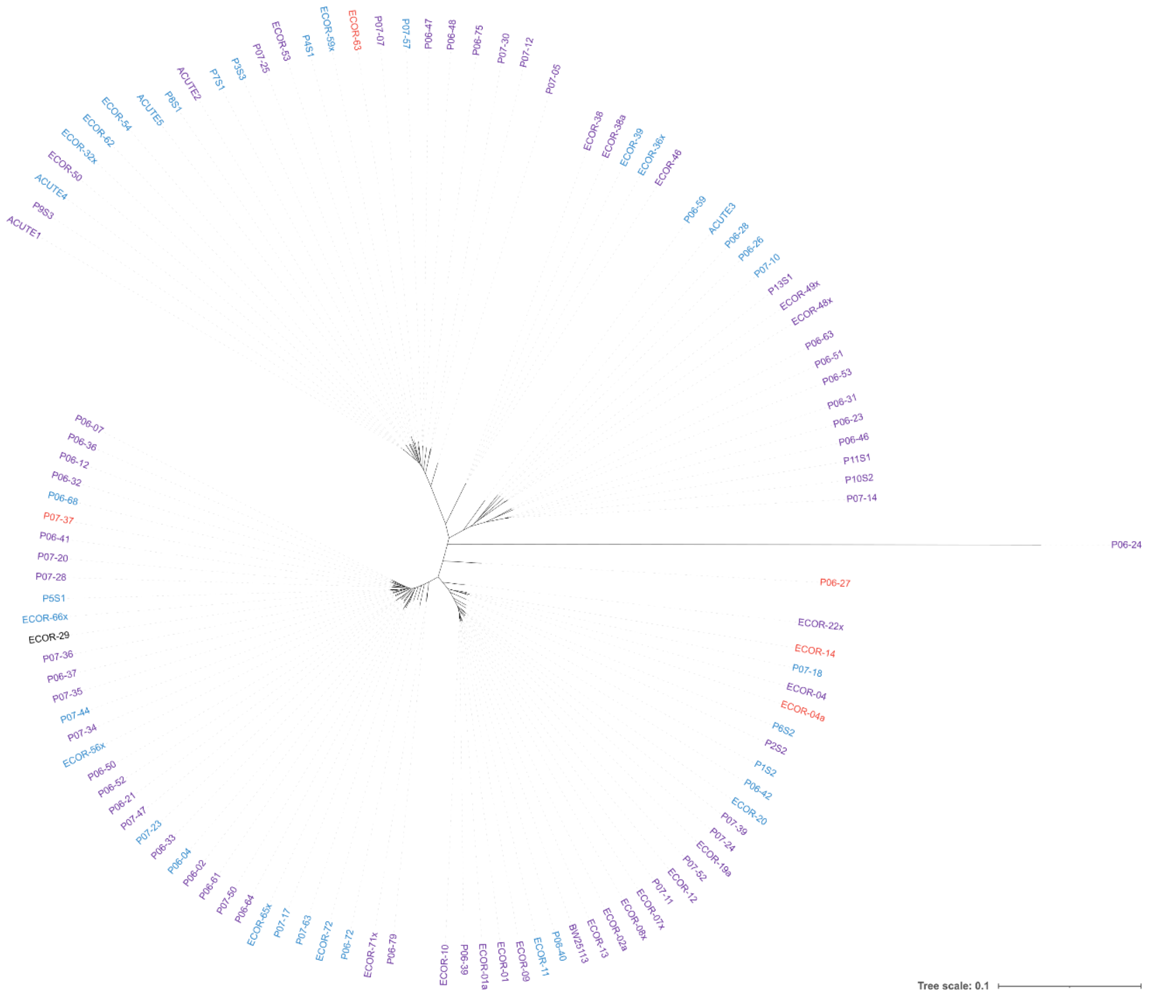

Figure S8. Conjugation efficiencies after 48 hours indicated on the RAxML phylogenetic tree. The labels of the strains are colored red for conjugation efficiencies between 10^0 and 10^{-1}, purple for conjugation efficiencies between 10^{-1} and 10^{-2}, blue for conjugation efficiencies between 10^{-2} and 10^{-3}, and black for conjugation efficiencies between 10^{-3} and 10^{-4}. Figure was created in iTOL 6.5.2.

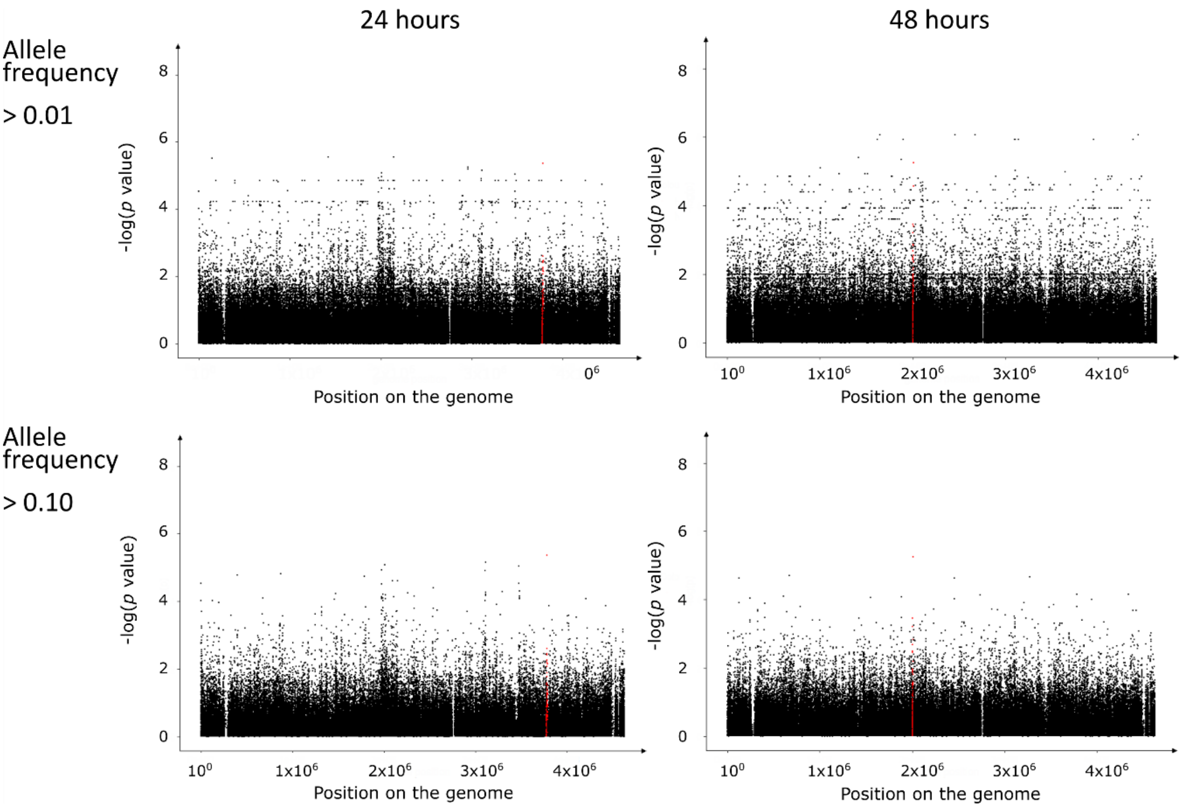

Figure S9. Manhattan plot of SNPs associated with the log-transformed conjugation efficiency after 24 hours and 48 hours using a linear mixed model. Horizontal lines are visible in the Manhattan plots in which SNPs with an allele frequency lower than 0.01 are filtered out, which may be attributed to lineage effects from population structure. These lines disappear when SNPs with an allele frequency lower than 0.10 are removed. SNPs within a region of 10 000 nucleotides surrounding the hit are marked in red.

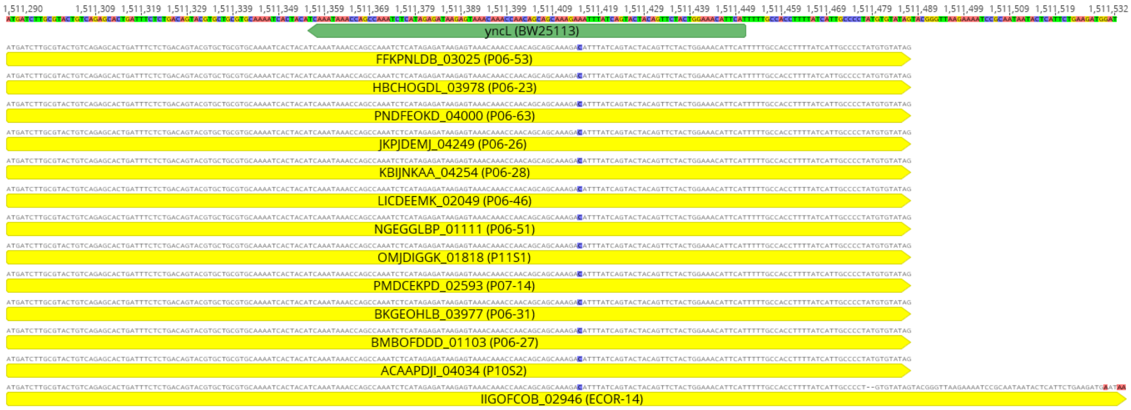

Figure S10. Alignment of all genes in group_9935 to the reference BW25113. The sequences in group_9935 were annotated by Prokka as hypothetical protein. However, they contain the sequence of *yncL* in the opposite direction. In this case, Prokka chose a different open reading frame compared to the annotation in BW25113. Compared to the reference, there is an A->C mutation at the 37th basepair of yncL. Alignment was done in Geneious.
